# Efficacy of a multicomponent binding agent against combined exposure to zearalenone and ochratoxin A in weaned pigs

**DOI:** 10.3389/fvets.2024.1357723

**Published:** 2024-03-06

**Authors:** Panagiotis Tassis, Jog Raj, Dimitrios Floros, Nikolaos Mittas, Niki Ntarampa, Hunor Farkas, Zoe Polizopoulou, Marko Vasilievic

**Affiliations:** ^1^Farm Animals Clinic, School of Veterinary Medicine, Aristotle University of Thessaloniki, Thessaloniki, Greece; ^2^Patent Co, DOO., Mišićevo, Serbia; ^3^Hephaestus Laboratory, Department of Chemistry, School of Science, International Hellenic University, Kavala, Greece; ^4^Diagnostic Laboratory, School of Veterinary Medicine, Aristotle University of Thessaloniki, Thessaloniki, Greece

**Keywords:** pigs, weaned, mycotoxins, zearalenone, ochratoxin A, adsorbent, mitigation, residues

## Abstract

**Introduction:**

The study aimed to evaluate the efficacy of a novel multicomponent substance against combined exposure to the mycotoxins zearalenone (ZEN) and ochratoxin A (OTA) in weaned piglets.

**Methods:**

In total, 60 piglets at the age of 28 days were equally allocated to four experimental groups (A–D), consisting of eight female and seven male piglets each (15 animals per group, for a total trial duration of 42 days). Animals from group A received typical weaner feed without mycotoxins or the test product [multicomponent mycotoxin detoxifying agent (MMDA)]. Group B animals received the same weaner feed contaminated with 0.992 mg ZEN/kg feed and 0.531 mg OTA/kg feed without the addition of the MMDA. Animals in group C received the same contaminated feed as group B with the addition of 1.5 g MMDA/kg feed, whereas group D received the same feed as group B with the inclusion of 3 g MMDA/kg feed. Clinical signs and performance parameters [body weight (BW), average daily weight gain (ADWG), and feed conversion ratio (FCR)] were evaluated, while mycotoxin residues were also assessed in the liver and kidney tissues.

**Results:**

Findings showed improved FCR in the group that received the greatest dose of the test product (3 g MMDA/kg feed) compared to the group that received the lower dose (1.5 g MMDA/kg feed). A few hematological and biochemical parameters were slightly altered, predominantly within normal limits. The residue analysis demonstrated a reduction of OTA in liver samples, a-ZEL in the liver and total tested samples, and a total of ZEN and metabolite contents in all samples of the group that received the greatest MMDA dose in comparison to the group that received the toxins without the addition of the test product.

**Discussion:**

Therefore, a positive effect of the MMDA at the greatest dosage regime on reducing bioavailability and tissue deposition of ZEN and OTA, with a particularly positive effect on FCR in weaned pigs, is suggested under concurrent ZEN and OTA exposure *in vivo*.

## 1 Introduction

A number of mycotoxins, as secondary metabolites of fungi detected in grains used for swine feed, has been suggested as harmful to the health and performance of swine (1). Particular mycotoxins from Fusarium species, such as zearalenone (ZEN), deoxynivalenol (DON), T2 toxin, and fumonisins (FB’s), and mycotoxins from Penicillium and Aspergillus species, such as aflatoxins (AFB’s) and ochratoxin A (OTA), are observed frequently in grains worldwide (2). The EU has set maximum or guidance limits for the above-mentioned mycotoxins. The guidance levels for compound feed contamination for piglets and gilts should not exceed 0.1 mg of ZEN/kg feed (0.25 mg of ZEN/kg feed for sows and fattening pigs) and 0.05 mg of OTA/kg feed, respectively (3).

Due to its estrogenic properties, ZEN binds to estrogen receptors (ERs), competing with 17-estradiol, and is typically associated with reproductive disorders in swine such as infertility, particularly in prepubertal gilts, leading to pseudopregnancy, increased embryo lethal resorptions, swollen edematous vulva, vaginal/rectal prolapse, and reduced litter size (due to fetal resorption and implantation failure) (4, 5). However, ZEN could also exhibit hepatotoxicity, hematotoxicity, immunotoxicity, and genotoxicity (6). Major derivatives of ZEN metabolism in pigs are α-zearalenol (α-ZEL), which is the predominant modified form in pigs with 3–100 times greater estrogenic activity than the parent toxin, and β-zearalenol (β-ZEL) (4).

Ochratoxin A is produced by several Aspergillus and Penicillium species and has an oral bioavailability of approximately 60% in pigs (7). After the absorption of the toxin from the gastrointestinal tract, binding to serum proteins (mainly albumin) follows (8, 9). ΟTA is enzymatically hydrolyzed to the less toxic ochratoxin-α (OTα) by intestinal microbes, whereas the cytochrome P450 system in the liver is associated with the hydroxylation of OTA to various forms of OTA-OH (10, 11). OTA is a major nephrotoxic agent, whereas it can also induce immunotoxic, genotoxic, neurotoxic, and teratogenic effects (7, 12). Pigs are significantly susceptible to the effects of OTA, and their tissue deposition is as follows: plasma>lung> kidney or heart>bile>liver>fat muscle (13, 14). OTA-associated clinical symptoms include reduced growth rate and feed efficiency with or without the reduction of feed consumption, along with hyperproteinemia and increased serum urea and creatinine, attributed to kidney function alterations (8, 15, 16). Particular *in vitro* studies demonstrated combined cytotoxic effects after ZEN and OTA administration and dose-dependent inhibition of cell viability on the HepG2 cell line, with OTA showing a greater cytotoxic effect than α-ZEL and ZEN (17–19).

The number of mycotoxins mitigating/detoxifying feed additives against ZEN or OTA effects *in vivo* has been previously reported (20–26). Such substances mainly include yeast products (e.g., *Saccharomyces cerevisiae boulardii* and *Trichosporon mycotoxinivorans*) (25), silymarin and other plant extracts (26–29), probiotics and microbes with degrading ability (e.g., *Bacillus licheniformis CK1*, or a strain of *Bacillus velezensis*, named A2), lactic acid bacteria (LAB), inorganic mineral adsorbents (e.g., tri-octahedral bentonite), and enzymes (e.g., laccases, carboxypeptidases, hydrolases, and amidases), as well as amino acids (selenomethionine and N-acetylcysteine), vitamins, and other organic synthetic adsorbents (30, 31).

Findings from *in vivo* tests with combined exposure to ZEN and OTA via feed to pigs are scarce (32–34). The study aimed to evaluate the efficacy of two different dosage regimes of a detoxifying multicomponent mycotoxin detoxifying agent (MMDA, Mycoraid, Patent Co, Misicevo, Serbia) containing modified zeolite (Clinoptilolite), *Bacillus subtilis*, *Bacillus licheniformis*, *Saccharomyces cerevisiae* cell wall, and silymarin in-feed against combined exposure to ZEN and OTA at levels exceeding the recommendation limits of the EU (3).

## 2 Materials and methods

### 2.1 Ethics statement

This study protocol was reviewed and approved according to the relevant regulatory standards by the Research Ethics Committee of the Aristotle University of Thessaloniki (AUTH) with approval number 122184/2021.

### 2.2 Animals and feed

A total of 60 weaned pigs at the age of 28 days were included in the study. Animals were purchased from a collaborating high-health status farrow-to-finish farm and were vaccinated a few days before purchase against enzootic pneumonia and porcine circovirus 2. Typical weaner feed (Table 1) was provided to the animals during the 42-day trial period (end of the study at 70 days of age). The trial was carried out in the collaborating experimental facility of the International Hellenic University (EL-54-BIOexp-50) in Sindos, Greece.

**Table 1:** Feed analysis and ingredients.

Ingredients	kg/t feed
Maize	564.13
Wheat	50
Soybean meal (47% CP)	252.02
Wheat bran	66.45
Soybean oil	13.35
Vitamin and mineral Premix 0.5%*	5
Limestone	24.82
Monocalcium Phosphate	12.97
Salt	3.5
L-lysine	3.49
DL-methionine	1.91
L-threonine	0.78
Chloride choline-50%	1.34
Magnesium oxide	0.11
Enzymes and antioxidants**	0.13
Chemical analysis	(%)
Dry matter	87.812
Crude protein	17.499
Crude fat	4.0
Ash	6.795
DProtein	16.329
Crude fiber	3.5
ADF	5.516
NDF	13.351
Ca	1.101
Total P	0.654
Lysine	1.2
Methionine + Cystine	0.811
Digestible Energy (MJ/kg)	13.988
Mycotoxin levels (feed of groups B, C, and D)	mg/kg feed
Zearalenone	0.992
Ochratoxin A	0.531

*Provided per kg of diet: 12000 IU vitamin A, 50 mcg 25-hydroxycholecalciferol, 9.96 mg vitamin E, 7.02 mg vitamin K3, 3 mg vitamin B1, 10.02 mg vitamin B2, 6 mg pantothenic acid, 6 mg vitamin B6, 40.02 mcg vitamin B12, 100 mg vitamin C, 35 mg niacin, 300 mcg biotin, 1.5 mg folic acid, 200 mg ferrous sulfate monohydrate, 90 mg copper sulfate pentahydrate, 60 mg manganese sulfate monohydrate, 100 mg zinc sulfate monohydrate, 2 mg calcium iodate, 300 mg sodium selenide, and 150 mg L- selenomethionine – selenium. **Provided per kg of diet: 1500 FYT 6-phytase, 80 U β-1,4-endoglucanase, 70 U β-1,3 (4)-endoglucanase, 270 U β-1,4-endoxylanase, 5,000 mg benzoic acid, 40,8 mg butylated hydroxytoluene, and 3.5 mg propyl gallate.

Pigs were allocated to four trial groups of 15 animals (eight female and seven male pigs/group). Group A received control feed without mycotoxins or the MMDA; group B served as positive controls and received a mycotoxin-contaminated diet with 992 μg ZEN/kg feed and 531 μg OTA/kg. Group C animals received the contaminated diet of group B with the inclusion of 1.5 g MMDA/kg feed, whereas group D animals received the same contaminated feed with 3 g MMDA/kg feed.

Spiked corn contaminated with 598 mg ZEN/kg and 890 mg OTA/kg was provided by the sponsor of the study for the contamination of trial feed. Analysis and standardization of contamination levels of spiked corn were performed at the sponsor’s laboratories with the use of LC–MS/MS. A standard procedure was performed for the preparation of trial feeds (27). The mixing of the MMDA and other feed materials was performed with very gentle mixing under minimum dust formation. Diets were manufactured in an appropriate production sequence, starting with group A control diets separated with a neutral meal mixed in between each of the diets and the production sequence, followed by group B feed, then group C, and finally group D. Subsamples of each experimental diet were collected for analysis according to the EC Regulation 152/2009 (35).

Feed analysis was performed in the Laboratory of Nutrition of the School of Veterinary Medicine of the AUTH. Feed analysis results are presented in Table 1. The final trial feed was further analyzed with LC–MS/MS for aflatoxins (B1, B2, G1, and G2), DON, ZEN, OTA, FB, T2, and HT2 in a collaborating laboratory (P. Androulakis & Partners Laboratories, Thermi, Greece). For the detection of AFB1, DON, ZEN, OTA, and T2 toxins, the method described by Ren et al. (36) was followed, with sequential extraction of homogenized samples by 80% (v/v) acetonitrile aqueous solution, followed by filtration and purification with an appropriate MycoSep SPE column (Romer Labs, Tulln, Austria) and reconstitution in a suitable LC/MS–MS solvent for analysis. All mycotoxin analysis results in trial feeds demonstrated the absence [< limit of quantification (LOQ) levels] of all but ZEN and OTA mycotoxins.

### 2.3 Clinical observations and performance parameters

Daily clinical evaluation was performed, and animals were examined for behavioral (reduced vigor) and eating disorders (anorexia), as well as signs of clinical mycotoxicosis associated with either ZEN or OTA ingestion, including the presence of cases of diarrhea, vomiting, vulval edema, rectal prolapse, and the occurrence of polyuria or polydipsia. To evaluate body condition and performance, animals were weighed weekly, and daily feed consumption was also calculated to assess body weight (BW), feed conversion ratio (FCR), as feed (kg): gain (kg) per time period, average daily weight gain (ADWG) values per period (0–21 and 21–42 days of the study), and for the whole study period.

### 2.4 Hematological and serum biochemical parameters

Three blood samplings were performed at the beginning (day 0), the end of the third week (day 21), and the end of the trial (day 42), resulting in a total of 180 blood samples for the assessment of biochemical and hematological parameters at the Diagnostic Laboratory of the School of Veterinary Medicine of AUTH. Blood samples were collected using BD Vacutainer tubes (BD-Plymouth, UK) and refrigerated for 24 h until centrifugation was carried out at 3000 rpm for 10 min for further serum collection and the analysis of biochemical parameters. In addition, blood samples were collected using microsample tubes EDTA K3E (Sarstedt, Nümbrecht, Germany) at the same time points for the assessment of hematological parameters.

Serum biochemical parameters were measured using an automated Vitalab flexor analyzer (Vital Scientific NV, Dieren, Netherlands). The tested parameters were total proteins, albumins, alanine aminotransferase (ALT), alkaline phosphatase (ALP), aspartate aminotransferase (AST), gamma-glutamyl transpeptidase (γ-GT), blood urea nitrogen (BUN), and creatinine (CRE).

Furthermore, the above-mentioned blood samples were tested with the use of an automated analyzer (Advia 120 hematology system, Bayer Diagnostics, Mfg Ltd., Dublin, Ireland) for the following hematological parameters: hematocrit (HCT), hemoglobin concentration (HGB), red blood cells (RBC), platelets (PLT), leucocytes (WBC), lymphocytes (LYM), neutrophils (NEUT), monocytes (MONO), eosinophils (EOS), basophils (BASO), mean corpuscular volume (MCV), mean corpuscular hemoglobin (MCH), mean corpuscular hemoglobin concentration (MCHC), erythrocyte distribution width (RDW), and mean platelet volume (MPV).

### 2.5 Residue analysis

At the end of the study, all animals were humanely euthanized with intravenous application of pentobarbital sodium (Repose®, Le Vet Beheer B.V., Netherlands) after introduction to anesthesia with a combination of ketamine (Ketabel®, Bela-Pharm GmbH and Co., Germany) and azaperone (Stresnil®, Elanco GmbH, Germany). One kidney and 100 g of liver tissue samples were collected from each animal and stored at -20°C before being freeze-dried. Tissues were further analyzed for mycotoxin residues of ZEN and OTA and their metabolites, including α- and β-ZEL, zearalanone (ZAN), α- and β- zearalanol (α-ZAL, β-ZAL) and OTα. An in-house LC–MS/MS method (Agilent 6,460 LC–MS/MS system) was performed with LOQ for ZEN and OTA at 0.4 μg/kg and for α-ZEL, β-ZEL, α-ZAL, β-ZAL, ZAN, and OTα at 4 μg/kg. The method was carried out with the use of internal standards (13C18) ZEN CRM BiopureTM (25 μg/mL) for zearalenone and its metabolites (13C20) OTA CRM BiopureTM (10 μg/mL) for OTA and OTα toxin. The recovery of more than 75% was recorded for all toxins. The method was linear from 0.4 to 8 μg/kg for ZEN and OTA, 4 to 80 μg/kg for α-ZEL, β-ZEL, α-ZAL, β-ZAL, ZAN, and OTα toxin. In brief, tissue samples were finely ground and thoroughly mixed using a blender. A 2-g test portion was removed for analysis. The samples were then extracted using a 10-ml extraction mixture (80% acetonitrile, 15% water, 5% formic acid) and by shaking the mixture in an orbital shaker at 200 rpm for 1 h at room temperature. After extraction, this portion was centrifuged at 4200 g for 5 min, and 7 mL of supernatant was removed and placed into another conical tube. The sample extract was cleaned by adding 2.8 g of MgSO4 and 0.7 g of NaCl to the supernatant and vortexed for 60 s. These tubes were centrifuged at 4200 g for 5 min. A 1 mL solution was removed from the supernatant and diluted with 250 μL of water. Further sample extract clean-up was performed on the Captiva EMR–Lipid cartridge (Agilent Technologies) (no cartridge conditioning is required): 1.25 mL of supernatant was passed through the cartridge (by gravity) and collected into a 15-ml centrifuge tube. When all the extract had passed through the cartridge, 400 μL of the extraction solvent was added to the cartridge and collected into the same centrifuge tube. The extract in the evaporator (CHRIST RVC 2–18 Cdplus) was evaporated at 1500 rpm under 40°C. Then, 500 μL of solvent for reconstitution (50% acetonitrile, 50% water containing 0.1% formic acid) was added to the evaporated sample and vortexed well. The prepared samples were filtered across a nylon membrane syringe (pore size of 0.22 μm) into a glass vial and vortexed. The samples were run on LC–MS/MS using analytical column Agilent ZORBAX Rapid Resolution HD 2.1*50 mm, 1.8 μm, and guard column ZORBAX Eclipse Plus C18, 2.1 mm, 1.8 μm, UHPLC guard column.

The test product administered in the study was an MMDA containing modified zeolite (clinoptilolite), *Bacillus subtilis*, *Bacillus licheniformis*, *Saccharomyces cerevisiae* cell wall, and silymarin (27).

### 2.6 Statistical analysis

Statistical analyses were conducted using the statistical software SPSS® Statistics (IBM) ver. 25 for the evaluation of mycotoxin residue results (non-repeated measurements) and the statistical programming language R for the rest of the parameters (repeated measurements) (37). For inferential purposes on the parameters referring to non-repeated measurements, the Kolmogorov–Smirnov test was used to assess whether the variables satisfied the normality assumption. Based on the derived findings, the one-way analysis of variance (ANOVA) was utilized to determine whether there was an overall effect of combined mycotoxins exposure on the response variables, followed by *post hoc* analysis through the Tukey or Duncan pairwise comparisons after the examination of the homogeneity of variance assumption. The non-parametric analog of one-way ANOVA, the Kruskal–Wallis test accompanied by Mann–Whitney pairwise comparisons, and the Bonferroni correction approach were used in the case of non-normal and highly skewed distributions. Finally, the investigation of the fixed effects of time and mycotoxin exposure (group) on the mean values for the rest of the parameters referring to repeated measurements was performed through the fitting of linear mixed-effects models (LMEMs). More specifically, an LMEM with both the main and interaction terms was fitted and tested against the model without the interaction term, while, in the case of a non-significant interaction term, the simpler model incorporating only the main effects was finally selected for inferential purposes. An alpha level of 0.05 was set for all statistical hypothesis testing procedures.

## 3 Results

### 3.1 Clinical observations and performance parameters

The absence of significant differences was detected among groups as regards BW for all comparisons and ADWG for each part and the total study period. Group C showed the lowest numerical ADWG and the greatest FCR for the total study period. On the contrary, animals in group D had significantly improved FCR values in the second part of the study (i.e., 21–42 days of age) in comparison to group B. The clinical evaluation of the animals suggested the absence of behavioral alterations, diarrhea, rectal prolapse, or anorexia signs during the course of the study, though variable edema of the external genitalia was present in groups B, C, and D, which received contaminated feed with or without the MMDA. The results on performance parameters are reported in Table 2.

**Table 2:** Performance parameters of the trial groups at each time point of the study (Mean ± SD)^#^.

Time point	Trial groups			
	A	B	C	D
BW (kg) 0d	8.29 ± 1.33	8.50 ± 1.05	8.56 ± 1.19	8.53 ± 1.18
BW (kg) 7d	10.62 ± 1.90	11.18 ± 2.30	10.71 ± 1.51	10.83 ± 1.39
BW (kg) 14d	13.73 ± 2.32	13.41 ± 1.93	13.19 ± 1.76	13.17 ± 1.63
BW (kg) 21d	16.14 ± 2.57	16.83 ± 2.53	15.99 ± 2.10	15.98 ± 1.98
BW (kg) 28d	19.07 ± 3.06	19.74 ± 3.05	18.39 ± 2.35	19.23 ± 2.27
BW (kg) 35d	23.84 ± 3.78	24.16 ± 3.74	22.65 ± 3.03	23.01 ± 2.38
BW (kg) 42d	28.14 ± 3.74	27.56 ± 4.02	26.76 ± 3.90	27.15 ± 2.38
FCR 0-21d	1.836 ± 0.012	1.644 ± 0.017	1.926 ± 0.031	1.843 ± 0.020
FCR 21-42d	1.783 ± 0.021^a^	1.942 ± 0.027^b^	1.973 ± 0.013^a^	1.794 ± 0.015^a^
FCR 0-42d	1.808 ± 0.022^a^	1.811 ± 0.069^ab^	1.954 ± 0.014^b^	1.814 ± 0.018^a^
ADWG (g) 0-21d	373.97 ± 71.74	396.51 ± 84.04	353.65 ± 60.25	364.13 ± 43.90
ADWG (g) 21-42d	571.43 ± 81.97	511.11 ± 88.20	513.02 ± 92.85	522.54 ± 81.96
ADWG (g) 0-42d	472,70 ± 67.90	453.81 ± 78.62	433.33 ± 73.24	443.33 ± 37.28

^a,b^Mean values with different superscripts in the same row differ significantly (*p* < 0.05). ^#^Group A: control feed without mycotoxins or the test product (MMDA); Group B: mycotoxin contaminated diet with 992 mg ZEN/kg feed and 531 mg OTA/kg feed; Group C: contaminated diet of Group B with the inclusion of 1.5 g MMDA/kg feed; and Group D: contaminated diet of Group B with the inclusion of 3 g MMDA/kg feed.

### 3.2 Hematological and biochemical parameters

Hematological analysis results (Table 3) did not suggest any significant deviation from normal limits. However, in five specific parameters (HCT, HGB, LYM, MONO, and MPV), significant general effects, without time X treatment interaction, were presented in comparisons between groups A and C (HCT, HGB, and MPV) or B and C (LYM and MONO). The mean values in group C were numerically reduced for all these parameters in comparison to groups A and B.

**Table 3:** The results of hematological parameters in all trial groups during the study (Mean ± SD).

	Trial groups			
	A	B	C	D
Parameter^∋^	Day 0			
HCT^*^ (%)	41.73 ± 1.78	40.23 ± 1.93	38.78 ± 1.66	37.74 ± 3.10
HGB^*^ (g/dl)	12.44 ± 0.58	12.12 ± 0.50	11.77 ± 0.58	11.38 ± 0.67
RBC (10^12^/l)	7.21 ± 0.31	6.89 ± 0.54	7.11 ± 0.39	6.76 ± 0.66
MCV (fl)	57.92 ± 2.37	58.65 ± 4.10	54.59 ± 2.28	55.92 ± 2.25
MCH (pg)	17.29 ± 1.06	17.67 ± 1.24	16.59 ± 0.77	16.92 ± 1.39
MCHC (g/dl)	29.82 ± 0.89	30.13 ± 0.56	30.38 ± 0.70	30.25 ± 1.74
RDW (%)	18.94 ± 3.61	18.50 ± 2.66	18.54 ± 2.47	19.14 ± 2.95
WBC (g/l)	14.76 ± 2.61	14.39 ± 2.47	15.88 ± 3.03	15.28 ± 3.35
NEUT (g/l)	5.37 ± 2.17	5.20 ± 1.99	6.57 ± 1.59	6.08 ± 3.43
LYM^*^ (g/l)	7.73 ± 1.47	7.64 ± 1.71	7.35 ± 2.19	7.18 ± 2.51
MONO^*^ (g/l)	0.12 ± 0.04	0.10 ± 0.05	0.11 ± 0.06	0.09 ± 0.05
EOS (g/l)	1.45 ± 0.44	1.40 ± 0.71	1.77 ± 1.24	1.86 ± 1.27
BASO (g/l)	0.08 ± 0.07	0.08 ± 0.03	0.08 ± 0.05	0.08 ± 0.02
PLT (g/l)	146.25 ± 38.88	162.30 ± 68.15	215.40 ± 117.87	205.69 ± 82.72
MPV^*^	14.63 ± 2.64	13.94 ± 1.92	12.58 ± 1.59	12.78 ± 1.43
	Day 21			
HCT^*^ (%)	40.10 ± 3.73	40.23 ± 4.24	36.53 ± 3.36	37.67 ± 4.21
HGB^*^ (g/dl)	11.42 ± 1.05	11.28 ± 0.99	10.70 ± 0.87	10.56 ± 1.02
RBC (10^12^/l)	6.81 ± 0.60	6.72 ± 0.64	6.49 ± 0.57	6.41 ± 0.75
MCV (fl)	59.00 ± 4.05	59.83 ± 2.77	56.29 ± 3.22	58.86 ± 3.53
MCH (pg)	16.79 ± 1.26	16.78 ± 0.78	16.51 ± 0.89	16.55 ± 1.1065
MCHC (g/dl)	28.48 ± 0.81	28.07 ± 0.85	29.34 ± 0.59	28.09 ± 0.78
RDW (%)	15.67 ± 0.96	16.70 ± 1.36	17.04 ± 0.97	16.60 ± 1.05
WBC (g/l)	19.02 ± 3.94	19.38 ± 4.09	17.21 ± 2.89	21.42 ± 6.50
NEUT (g/l)	7.85 ± 2.15	6.63 ± 1.38	6.98 ± 2.03	9.17 ± 2.66
LYM^*^ (g/l)	9.79 ± 3.01	11.31 ± 2.78	9.20 ± 2.49	10.82 ± 4.78
MONO^*^ (g/l)	0.69 ± 0.46	0.78 ± 0.28	0.50 ± 0.26	0.67 ± 0.32
EOS (g/l)	0.45 ± 0.31	0.35 ± 0.13	0.34 ± 0.17	0.46 ± 0.12
BASO (g/l)	0.16 ± 0.10	0.18 ± 0.10	0.13 ± 0.04	0.23 ± 0.30
PLT (g/l)	263.08 ± 119.09	270.17 ± 77.79	269.57 ± 84.33	277.92 ± 108.23
MPV^*^	12.58 ± 1.44	11.55 ± 1.34	11.20 ± 1.34	12.28 ± 1.44
	Day 42			
HCT^*^ (%)	38.34 ± 3.63	40.61 ± 2.93	36.58 ± 9.30	39.19 ± 2.94
HGB^*^ (g/dl)	10.90 ± 0.78	11.63 ± 0.70	10.13 ± 3.06	11.03 ± 0.57
RBC (10^12^/l)	6.47 ± 0.59	6.93 ± 0.55	6.22 ± 1.91	6.66 ± 0.45
MCV (fl)	59.39 ± 4.32	58.79 ± 3.90	57.87 ± 3.27	58.90 ± 3.16
MCH (pg)	16.91 ± 0.98	16.85 ± 1.01	16.37 ± 0.90	16.60 ± 1.07
MCHC (g/dl)	28.53 ± 1.09	28.71 ± 0.75	28.29 ± 1.03	28.19 ± 1.92
RDW (%)	17.81 ± 1.12	17.96 ± 0.96	18.14 ± 0.83	17.46 ± 0.89
WBC (g/l)	14.80 ± 3.48	15.99 ± 1.90	13.62 ± 4.70	15.14 ± 2.53
NEUT (g/l)	6.38 ± 2.03	7.01 ± 1.97	6.03 ± 2.24	8.10 ± 2.69
LYM^*^ (g/l)	7.20 ± 1.88	7.65 ± 1.60	6.53 ± 2.42	5.94 ± 1.67
MONO^*^ (g/l)	0.65 ± 0.29	0.58 ± 0.26	0.41 ± 0.23	0.53 ± 0.25
EOS (g/l)	0.40 ± 0.09	0.53 ± 0.18	0.48 ± 0.27	0.44 ± 0.18
BASO (g/l)	0.10 ± 0.04	0.12 ± 0.06	0.09 ± 0.04	0.09 ± 0.02
PLT (g/l)	217.50 ± 84.55	240.25 ± 144.72	270.90 ± 68.92	239.57 ± 60.59
MPV^*^	13.54 ± 3.15	12.78 ± 1.90	11.60 ± 1.87	13.00 ± 1.43

^*^Main effect without time x treatment effect: p-values HCT Group A vs. Group C = 0.024 and Group B vs. Group C = 0.024; *p*-values HGB Group A vs. Group C = 0.031 and Group B vs. Group C = 0.033; p-values LYM Group B vs. Group C = 0.042; p-values MONO Group B vs. Group C = 0.042; p-values MPV Group A vs. Group C = 0.001 and Group B vs. Group C = 0.051. ^#^Group A: control feed without mycotoxins or the test product (MMDA); Group B: mycotoxin contaminated diet with 992 mg ZEN/kg feed and 531 mg OTA/kg feed; Group C: contaminated diet of Group B with the inclusion of 1,5 g MMDA/kg feed; and Group D: contaminated diet of Group B with the inclusion of 3 g MMDA/kg feed. ^∋^Normal reference values (38, 39): HCT: 33–45; HGB: 10.8–14.8; RBC: 5.8–8.1; MCV: 50–65; MCH: 17–21; MCHC: 30–35; RDW: 14.3–26; WBC: 10–22; NEUT: 3–17.4; LYM: 7.7–20.4; MONO: 0.6–3.4; EOS: 0.1–2.3; BASO: 0.1–0.3; PLT: 220–620; MPV: 7.4–16.5.

The results of serum biochemical parameters for all groups at specific time points are demonstrated in Table 4. ALP value alterations were observed due to normal animal growth in the course of the study. Significant differences among groups at each time point of the study fell within the normal limits range (e.g., CRE at all sampling time points) or very close to the bordering minimum or maximum levels (e.g., albumins at the 2nd and 3rd sampling time points).

**Table 4:** Serum biochemical parameters in all trial groups and time points of sampling (Mean ± SD)^#^.

	Trial groups			
	A	B	C	D
Parameters^∋^	Day 0			
Total Proteins (g/dl)	5.47 ± 0.39^a^	5.13 ± 0.23^b^	4.94 ± 0.45^b^	4.90 ± 0.27^b^
Albumins (g/dl)	3.68 ± 0.26^a^	3.41 ± 0.21^b^	3.72 ± 0.35^a^	3.78 ± 0.19^a^
ALT^¶^ (U/l)	45.13 ± 8.18	41.50 ± 5.52	39.79 ± 8.67	38.87 ± 4.32
AST^¶^ (U/l)	80.67 ± 31.97 ^a^	63.21 ± 21.21 ^ab^	63.14 ± 30.88^b^	56.60 ± 15.25^b^
ALP (U/l)	1311.87 ± 314.93^a^	1090.00 ± 180.12^ab^	990.14 ± 255.01^b^	968.13 ± 277.25^b^
γ-GT (U/l)	19.93 ± 8.44^a^	16.93 ± 9.32^ab^	14.43 ± 6.04^ab^	18.60 ± 8.32^b^
BUN^¶^ (mmol/l)	6.73 ± 2.96^ab^	5.50 ± 2.59^a^	8.64 ± 3.73^b^	7.80 ± 3.43^b^
CRE (mg/dl)	1.21 ± 0.13^a^	1.01 ± 0.15^b^	0.89 ± 0.12^c^	0.76 ± 0.11^d^
	Day 21			
Total Proteins (g/dl)	5.00 ± 0.71^a^	5.79 ± 0.41^b^	5.54 ± 0.42^bc^	5.33 ± 0.38^c^
Albumins (g/dl)	3.59 ± 0.49^a^	4.07 ± 0.35^b^	3.95 ± 0.25^b^	3.84 ± 0.20^b^
ALT^¶^(U/l)	42.87 ± 8.62	43.20 ± 8.94	41.08 ± 8.58	45.46 ± 6.75
AST^¶^ (U/l)	51.53 ± 15.36^a^	73.87 ± 26.12^bc^	79.38 ± 28.80^c^	64.08 ± 27.24^ac^
ALP (U/l)	699.93 ± 179.47^a^	1342.13 ± 307.59^c^	1356.46 ± 341.26^c^	1089.46 ± 255.13^b^
γ-GT (U/l)	21.67 ± 9.91^a^	23.00 ± 13.04^b^	27.46 ± 16.84^b^	22.00 ± 6.12^a^
BUN^¶^ (mmol/l)	5.80 ± 2.01^a^	5.73 ± 2.34^a^	8.15 ± 3.05^b^	5.23 ± 1.69^a^
CRE (mg/dl)	0.77 ± 0.11^a^	1.01 ± 0.13^b^	1.04 ± 0.13^b^	0.97 ± 0.08^b^
	Day 42			
Total Proteins (g/dl)	5.37 ± 0.46^a^	5.93 ± 0.41^b^	5.74 ± 0.27^a^	5.60 ± 0.19^a^
Albumins (g/dl)	4.05 ± 0.39	4.25 ± 0.30	4.18 ± 0.25	4.19 ± 0.29
ALT^¶^(U/l)	47.67 ± 8.61	48.20 ± 7.81	40.13 ± 9.23	46.47 ± 8.78
AST^¶^ (U/l)	48.93 ± 11.62^a^	58.80 ± 16.52^ab^	72.13 ± 44.01^b^	67.27 ± 33.02^ab^
ALP (U/l)	506.00 ± 129.03^a^	671.67 ± 214.68^b^	644.80 ± 316.33^ab^	608.33 ± 186.24^ab^
γ-GT (U/l)	20.67 ± 12.28	20.00 ± 10.50	17.28 ± 8.31	19.67 ± 4.65
BUN^¶^ (mmol/l)	5.67 ± 2.55^ab^	5.87 ± 3.46^a^	5.47 ± 2.33^a^	7.73 ± 3.49^b^
CRE (mg/dl)	0.75 ± 0.09^a^	0.95 ± 0.11^b^	0.98 ± 0.13^b^	1.04 ± 0.16^b^

^a,b,c,d^Mean values with different superscripts in the same row differ significantly (*p* < 0.05) with treatment x time interaction present. ^¶^Parameters with significant main effects among groups (*p* < 0.05) without treatment x time interaction present: ALT p values: Group A vs. Group C: *p* = 0.012; Group B vs. Group C: *p* = 0.032. ^#^Group A: control feed without mycotoxins or the test product (MMDA); Group B: mycotoxin contaminated diet with 992 mg ZEN/kg feed and 531 mg OTA/kg feed; Group C: contaminated diet of Group B with the inclusion of 1,5 g MMDA/kg feed; and Group D: contaminated diet of Group B with the inclusion of 3 g MMDA/kg feed. ^∋^Normal reference values (39, 40): Total proteins: 4.4–7.4; albumins: 1.9–3.9; alanine aminotransferase (ALT): 8–46; aspartate aminotransferase (AST): 21–94; alkaline phosphatase (ALP): 142–891, gamma-glutamyl transpeptidase (γ-GT): 0–82, blood urea nitrogen (BUN): 2.9–8.89; creatinine (CRE): 0.76–1.95.

### 3.3 Tissue residues

Residue analysis demonstrated reduced values of OTA, ZEN, and metabolites in the liver and kidneys of animals receiving MMDA. Results are presented in Table 5 and include comparisons among groups B, C, and D since group A mean values for all toxins and metabolites were below LOQ. Moreover, the results of ZAN, α- and β- ZΑL, and ΟΤα were < LOQ in all samples from all trial groups tested.

**Table 5:** Residues of ZEN, OTA, α-ZEL, and β-ZEL in the liver and kidney samples of the groups B, C, and D at the end of the trial (Mean ± SD)^¶^.

Trial groups			
Tissues samples	B	C	D
OTA (μg/kg)			
Liver samples	351.8 ± 116.89^a^	342.27 ± 108.47^a^	255.80 ± 68.2^b^
reduction vs. group B (%)^∋^		−2.71%	27.29%
Kidney samples	706.73 ± 251.52	628.4 ± 215.31	617 ± 172.55
reduction vs. group B (%)		−11.08%	12.7%
All samples (liver and kidney)	529.27 ± 264.04	485.33 ± 221.89	436.4 ± 224.41
reduction vs. group B (%)		−8.3%	−17.55%
ZEN (μg/kg)			
Liver samples	6.33 ± 2.81	6.18 ± 2.41	5.57 ± 2.14
reduction vs. group B (%)		−2.37%	−12%
Kidney samples	12.51 ± 7.42	10.12 ± 4.36	9.72 ± 4.37
reduction vs. group B (%)		−19.1%	−22.3%
All samples (liver and kidney)	9.42 ± 6.34	8.15 ± 4.00	7.65 ± 3.98
reduction vs. group B (%)		−13.48%	−18.79%
a-ZEL (μg/kg)			
Liver samples	16.57 ± 7.03^a^	15.53 ± 4.68^a^	10.1 ± 2.55^b^
reduction vs. group B (%)		−6.28%	−39.05%
Kidney samples	18.18 ± 6.89^a^	15.29 ± 5.26^ab^	12.98 ± 5.68^b^
reduction vs. group B (%)		−15.9%	−28.6%
All samples (liver and kidney)	17.37 ± 6.89^a^	15.41 ± 4.89^a^	11.54 ± 4.56^b^
reduction vs. group B (%)		−11.28%	−33.56%
b-ZEL (μg/kg)			
Liver samples	7.94 ± 2.33	7.2 ± 1.4	7.16 ± 1.67
reduction vs. group B (%)		−9.32%	−9.82%
Kidney samples	5.06 ± 2.62	4.19 ± 0.42	4.1 ± 0.43
reduction vs. group B (%)		−17.19%	−20.95%
All samples (liver and kidney)	6.5 ± 2.84	5.69 ± 1.84	5.63 ± 1.96
reduction vs. group B (%)		−12.46%	−13.39%
Total ZEN, a-ZEL and b-ZEL (μg/kg)			
Liver samples	30.84 ± 9.94^a^	28.92 ± 5.90^ab^	22.59 ± 5.40^b^
reduction vs. group B (%)		−6.23%	−26.75%
Kidney samples	34.49 ± 16.77	28.3 ± 10.26	25.03 ± 10.16
reduction vs. group B (%)		−17.95%	−27.43%
All samples (liver and kidney)	32.66 ± 13.67^a^	28.61 ± 8.23^ab^	23.81 ± 8.09^b^
reduction vs. group B (%)		−12.4%	−27.1%

^¶^All samples from Group A [animals that received control feed without mycotoxins or the test product (MMDA)] had values < Limit of Quantitation of all mycotoxins and metabolites tested. ^a,b^Mean values with different superscripts in the same row differ significantly (*p* < 0.05). ^∋^Percentage differences presented refer to the numerical reduction (−) of the mean values of each toxin only in comparison with the positive control group B. ^#^Group B: mycotoxin-contaminated diet with 992 mg ZEN/kg feed and 531 mg OTA/kg feed; Group C: contaminated diet of group B with the inclusion of 1.5 g of the test product (MMDA)/kg feed; and Group D: contaminated diet of group B with the inclusion of 3 g of MMDA/kg feed.

In group D, a significant reduction of mean values (reduction by 27.29%) of OTA residues in liver samples was reported in comparison to group B. Residues of ZEN (parent form) and β-ZEL mean values were not significantly altered. However, α-ZEL, the predominant ZEN metabolite in swine, was significantly reduced in liver samples of group D when compared with group B. A similar reduction was present in group D when comparing the total residues of that metabolite in both liver and kidney samples combined. A greater than 26% reduction of combined ZEN and metabolites residue mean values in both liver and kidney samples were observed in group D when compared with group B (26.75% reduction in liver samples, 27.43% in kidney samples, and 27.1% in both tissues of group D). Nevertheless, a numerical reduction of all detected parent mycotoxins and metabolites residue mean values could be reported in group C, which received the lower MMDA dosage level.

## 4 Discussion

The results of our study provide evidence of the ability of the MMDA at the level of 3 g/kg feed to reduce adsorption, metabolism, and tissue deposition of ZEN and OTA in weaned pigs that received feed with significant contamination levels of ZEN and OTA for a time period of 42 days. The reduction of tissue residues of ZEN, metabolites, and OTA in trial group D and the improvement of FCR values in the second half of the study support the aforementioned ability of the MMDA in pigs under combined ZEN and OTA exposure *in vivo*.

The findings in the respective literature regarding the effects of combined *in vivo* exposure to ZEN and OTA in pigs are scarce. Lusky et al. (41) introduced combined contaminated feed (100 μg OTA and 250 μg ZEN/kg feed, respectively) to 50–60 kg pigs for 90 days and reported that OTA metabolism or excretion is influenced by the simultaneous administration of ZEN. Moreover, the insignificant effect of combined exposure to the final BW of animals was highlighted, as also observed in our results. In other research efforts with the use of similar to our study concentrations of ZEN and OTA, but with single—not combined—administration of each mycotoxin, it was shown that after feeding weaned gilts with 1.04 mg ZEN/kg feed for 35 days (42) or after ingestion of contaminated feed with 500 μg OTA/kg for 15 days in growers (14), a similar absence of significant final BW alterations was present. However, our results showed numerical differences in BW gain from the midpoint to the end of the study. An approximate increase in mean body weight during the second part of the study (21–42 days) was present in all trial groups and reached 12 kg in the control group, whereas the BW increase for the same time period in group B was 10.73 kg, in group C was 10.77 kg, and in group D was 11.17 kg. The numerical improvement of 440 g mean body weight in group D compared with group B could be associated with MMDA incorporation in the feed of group D, supporting an overall positive, productive outcome. On the other hand, improved FCR values of group D in the second part of the study (21–42 study days) in comparison to group B could also be an indication of a positive effect of the MMDA on the performance of animals in group D.

ZEN undergoes rapid and extensive absorption of up to 80–85% in pigs, and significant parts of its metabolic route are carried out predominantly in the liver and intestine, whereas the parent toxin and its metabolites are eliminated relatively slowly from the tissues by enterohepatic circulation (4, 5, 43). Therefore, the liver is the major organ of ZEN distribution (6), which explains the differences in the mean levels of residue findings among liver and kidney samples in our study. Hydroxylation of ZEN to α-ZEL in pig liver microsomes is considered an activation process since the estrogenic potency of α-ZEL is significantly greater than the parent toxin in swine due to its greater affinity for estrogen receptors, whereas β-ZEL represents a metabolite with reduced estrogenic activity (43). Our findings on the distribution of ZEN and metabolite levels in liver tissue are in agreement with Zöllner et al. (44), who reported predominantly α-ZEL and, to a lesser extent, β-ZEL and ZEN in liver samples after feeding ZEN-contaminated oats to growers. A ratio of 2.5:1 in α-:β-ZEL level was demonstrated in the liver samples of that study. Our findings showed a quite comparable α-:β-ZEL ratio of 2.09 in group B, and 2.16 in group C, but only 1.4 in group D due to the significantly reduced α-ZEL residue values. Such findings provide clear evidence of reduced ZEN uptake and metabolism toward α-ZEL. The reduction of the specific metabolite in group D was observed to be the greatest in the present study, reaching approximately 39.05% compared to group B.

Findings on OTA residues at greater concentrations in the kidneys than in the liver in our study are in agreement with a previous study (41), which reported that greater OTA residues in kidneys are observed after combined ZEN and OTA ingestion by pigs rather than OTA-alone introduction in feed. The liver is the tissue with greater OTA residues after the kidneys when combined ZEN and OTA feed ingestion occurs in pigs. Reduction of OTA residues in those significant reservoirs of the toxin was present in our study, being statistically significant in the liver samples of group D, supporting a mitigating effect of the MMDA at the dosage level used in that group.

Studies with the ingestion of greater ZEN concentrations of 1.22 mg ZEN/kg feed for 28 days (31) or 1.3 mg ZEN/kg feed (45) for 24 days reported a significant increase in biochemical parameters (BUN, CRE, AST, ALT, and γ-GT values), suggesting possible liver and kidney damage, which was deteriorated with the addition of either Vitamin C or a montmorillonite clay adsorbent. Our findings after the ingestion of lower levels of ZEN along with OTA than the above-mentioned studies cannot support a heavy liver and kidney dysfunction since all tested parameters were within or close to the normal limits. On the other hand, our results on the 21st day of the study demonstrate a significant reduction of ALP and a numerical reduction of the parameter at the end of the study in group D when compared with group B, thus supporting a possible liver protective effect of the MMDA after 3 weeks of continuous consumption of contaminated feed. In addition, the mean values of total proteins and albumins at the same time point in groups B, C, and D were greater than the control group, thus being in contrast with *in vitro* findings of Abid-Essefi et al. (46) on the ability of ZEN to inhibit protein synthesis in Vero and Caco-2 cells, but providing ground for discussion on the possible enhancement of efficiency of protein conversion induced by ZEN metabolic effects as suggested by Su et al. (31).

Apart from the above-mentioned biochemical parameter alterations, Jiang et al. (45) supported an effect of 1.3 mg ZEN/kg feed on the PLT and HGB mean values of weaned pigs. Our findings present an absence of effect on PLT values, whereas a main effect (without time x treatment interaction) of the toxins on HGB values was detected in group C in comparison to both groups A and B. However, a significant hematotoxic effect after combined ZEN and OTA exposure through feed in our study cannot be supported. Differences in findings among studies are probably related to interactions among the two toxins used in our study instead of a single ZEN administration. Moreover, the alterations observed on HCT and HGB levels in the aforementioned study were discussed as transient and reversible after 14 days of continuous mycotoxin ingestion, thus supporting an explanation of the main effect observed on HCT and HGB in our study, even though all mean values were within the normal range.

The importance of considering the mixed effects of multi-mycotoxin contamination on swine health and productivity is undeniable. As previously reported, mycotoxin-detoxifying agents containing more than one component to alleviate the toxic effects of mycotoxins have shown more benefits in comparison to those with single components (22). The MMDA in the present study, as a combination of multiple mycotoxin-detoxifying agents, has been previously reported as capable of reducing the negative outcome of combined exposure to ZEN and T2 in weaned pigs (27). It was demonstrated that the MMDA had a dose-dependent affinity for ZEN and, to a lesser extent, for T2 toxin, providing a greater effect at the inclusion level of 3 g/kg feed. Quite similarly, in the present study, an affinity to both ZEN and OTA is demonstrated at the same concentration level of 3 g/kg feed.

The results of the MMDA inclusion in the feed are based on the combined effects of its ingredients. The combined effect of the adsorbing ability of modified zeolite (Clinoptilolite) and the detoxification effects of *Bacillus subtilis*, *Bacillus licheniformis*, *Saccharomyces cerevisiae* cell wall, and silymarin could be suggested as the primary causes for the reduction of ZEN and OTA bioavailability and tissue deposition observed in our study. Modified clay materials, such as zeolite, have been proven efficacious in the past decades in ameliorating ZEN and OTA effects, whereas various Bacillus strains, such as *B. subtilis, B. licheniformis*, *B. amyloliquefaciens*, *B. cereus*, and *B. velezensis*, have also been suggested as significant biological detoxification agents for ZEN and OTA (20, 21). The biological detoxification process includes the adsorption of the mycotoxin onto the walls of the microbial cells or its degradation by microbial secretases. The adsorption capacity of the vast majority of Bacillus spp. strains are of reduced importance when compared with their degradation effects due to secretases (21). On the other hand, the ability of *Saccharomyces cerevisiae* to bind ZEN to its cell walls has been reported, whereas the importance of the amount of β-D-glucan in the cell walls of *S. cerevisiae* on mycotoxins binding ability and a correlation between cell wall surface area and ZEN and OTA removal ability has also been highlighted (47, 48). Silymarin is a liver protective agent that is capable of reducing the negative effects of AFB1 on the performance of broiler chicks (49), whereas in rats, dietary silymarin supplementation at 100, 200, and 500 mg/kg (50) protected from ZEN-induced hepatotoxicity and reproductive toxicity. The latter improvement was attributed to the observed improvement of antioxidant capacity, the regulation of genes related to ZEN metabolism, hormone synthesis, protein synthesis, and ABC transporters in the tissues.

## 5 Conclusive remarks

The results of the present *in vivo* study on weaned pigs with combined exposure to significant concentrations of ZEN and OTA demonstrated that the tested MMDA at a 3 g/kg feed dosage level can reduce the residues of ZEN and its major metabolites and OTA in the liver and improve FCR values after 21 days of ingestion of contaminated diets. Therefore, it can be suggested that MMDA should be considered as a mitigation strategy to address mixed ZEN and OTA challenges in pigs.

## Data availability statement

The original contributions presented in the study are included in the article/supplementary material, further inquiries can be directed to the corresponding author/s.

## Ethics statement

The animal study was approved by the Research Ethics Committee of the Aristotle University of Thessaloniki. The study was conducted in accordance with the local legislation and institutional requirements. Written informed consent was obtained from the owner of the farm of origin of the trial animals.

## Author contributions

PT: Conceptualization, Data curation, Investigation, Methodology, Writing – original draft, Writing – review & editing. JR: Conceptualization, Resources, Writing – original draft, Writing – review & editing. DF: Investigation, Methodology, Writing – review & editing. NM: Data curation, Formal analysis, Writing – original draft, Writing – review & editing. NN: Investigation, Writing – review & editing. HF: Investigation, Resources, Writing – original draft, Writing – review & editing. ZP: Investigation, Methodology, Writing – review & editing. MV: Funding acquisition, Supervision, Writing – review & editing.
